# Incidence neurosensory deficit in mandibular fractures

**DOI:** 10.6026/97320630019725

**Published:** 2023-06-30

**Authors:** Sneha Alladi, Pradeep Dhasarathan, Hemavathy O.R Muralidoss, Krishnan Murugesan, Vedha Vivigdha

**Affiliations:** 1 Department of Oral and Maxillofacial Surgery, Saveetha Dental College and Hospitals, Saveetha Institute of Medical and Technical Sciences, Saveetha University, Chennai, India

**Keywords:** Neurosensory deficit, Mandibular fractures, Lower Lip paraesthesia, Inferior alveolar nerve, mental nerve

## Abstract

Sensory disturbances in the inferior alveolar nerve (IAN) prior to treatment can be attributed to various factors, including the site, type of fracture, and fracture displacement. Therefore, it is of interest to assess the incidence of inferior alveolar nerve injuries associated with mandibular fractures before and after surgical treatment. Group A consisted of patients with inferior alveolar nerve paresthesia before treatment, while Group B consisted of patients with inferior alveolar nerve paresthesia after treatment. A significant difference was observed between the two groups, with a p-value of 0.031 (p <0.05) with the overall incidence of IAN deficit was 57.33% before treatment and 61.33% after treatment. These findings highlight the importance of promptly identifying and managing IAN injuries to minimize long-term consequences.

## Background:

Mandibular fractures represent a common and significant component of maxillofacial trauma, with the mandible being the most frequently fractured bone in the facial skeleton [1, 2]. It can lead to various complications, such as occlusal derangement, airway obstruction, and paresthesia. Among the potential complications of mandibular fractures, injuries to the inferior alveolar nerve (IAN) have garnered considerable attention due to their clinical implications and impact on patient outcomes [3]. Neurosensory deficits associated with the inferior alveolar nerve include paresthesia, hyperesthesia, and loss of tooth vitality. Paresthesia and loss of tooth vitality are the most commonly observed neurosensory disturbance in mandibular fractures, with paresthesia of the lower lip being the most prominent. Sensory disturbances in the inferior alveolar nerve before treatment can be attributed to various factors, including the site, type of fracture, and fracture displacement [4]. Postoperatively, nerve paresthesia is frequently observed neurosensory disturbance, resulting from injury to the inferior alveolar nerve caused by traction, manipulation, and iatrogenic factors. Although these sensory disturbances are often transient, they can cause considerable discomfort and functional limitations during healing. Therefore, assessing the incidence of inferior alveolar nerve injuries associated with mandibular fractures before and after surgical treatment is of interest.

##  Materials and Methods:

This study was conducted at a private dental college's Department of Oral and Maxillofacial Surgery. The study involved a cohort of 150 patients diagnosed with mandibular fractures. The study period included from November 2021 to November 2022. Ethical approval for the study was obtained from the Institutional Research Board Committee. All the demographic data was collected from each patient. The inclusion criteria were patients diagnosed with mandibular fractures that extended from the mental foramen to the mandibular foramen, patients who had unilateral fractures, and patients who underwent surgical intervention for mandibular fractures. Exclusion criteria encompassed patients under the age of 18 (pediatric fractures), patients with multiple mandibular fractures, unilateral fractures of the condyle, fractures of the symphysis, pathological fractures, and Patients with a history of mandibular fractures or existing neurosensory deficit. The groups consisted of patients with inferior alveolar nerve paresthesia. Group A consisted of patients with inferior alveolar nerve paresthesia before treatment, while Group B consisted of patients with inferior alveolar nerve paresthesia after treatment. The paresthesia was assessed using a light touch test, which involved gently touching the lip with a cotton wisp while the patient's eyes were closed. This test evaluated the detection threshold for tactile stimulation.

## Surgical Procedure:

Under general anesthesia with standard aseptic protocols, an injection of 1:200000 adrenalines was administered. The surgical approaches were intra-oral when there was an absence of existing laceration. A full-thickness mucoperiosteal flap was elevated. Mental nerve was identified and carefully dissected. Fracture segments were identified and reduced using mini plates. The closure was done with simple interrupted sutures using 3-0 polyglactin.

## Statistical analysis:

All the acquired data were entered into Excel Spreadsheet (Microsoft, USA). Data were analyzed using SPSS version 21 (IBM Corp., Armonk, NY, USA). The data were normally distributed and therefore non-parametric tests were performed. The data were expressed as Mean and SD. McNemar Test was performed between the groups. A two-sided p-value <0.05 was considered statistically significant.

## Results:

**Figure 1:**
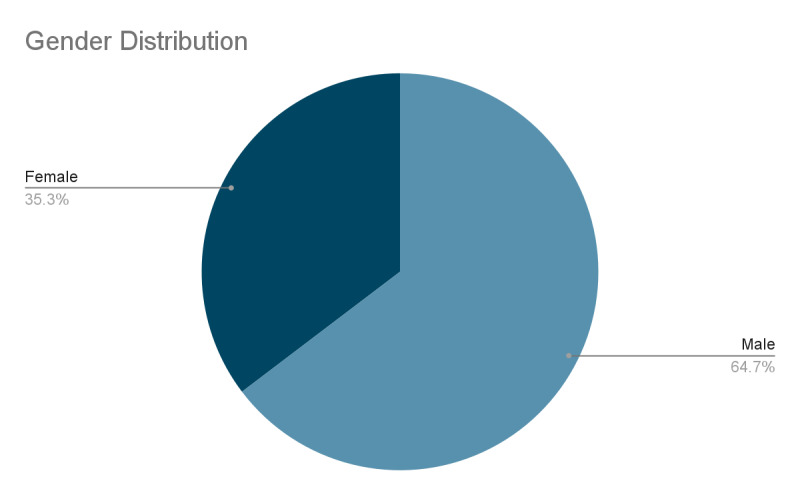
Gender Distribution

**Figure 2:**
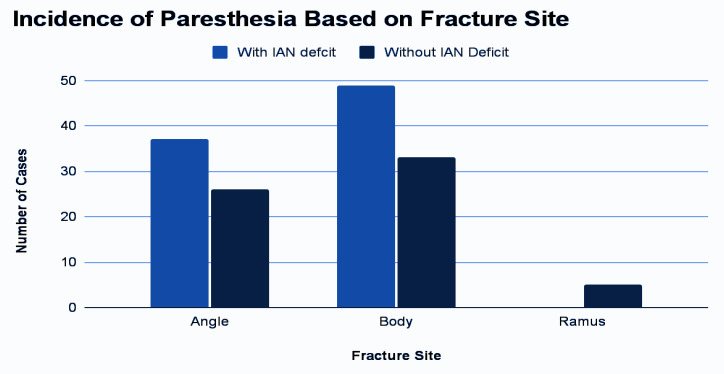
Incidence of Paresthesia Based on Fracture Site Inferior Alveolar Nerve (IAN).

A total of 150 patients with mandibular fractures were included in the study. The mean age was 34.3 years, and the majority of patients were male (56%) (Table 1 and Figure 1). Assaults were the leading cause of mandibular fractures (38%), followed by road traffic accident (36%) and falls (26%). This image represents a pie chart depicting the total distribution of age among the study samples considered within the limits of the study. The chart shows that 64.67% of participants were female, while 35.33% were male. The most common fracture site was the body of the mandible (54.67%), followed by the angle (42%) and the ramus (3.33%). Among these, the ramus fracture did not show any inferior alveolar nerve (IAN) deficit. However, IAN deficit was observed in 37 patients with angle fracture and 49 patients with a body of the mandible fracture before treatment (Table 2) (Figure 2).

**Figure 3:**
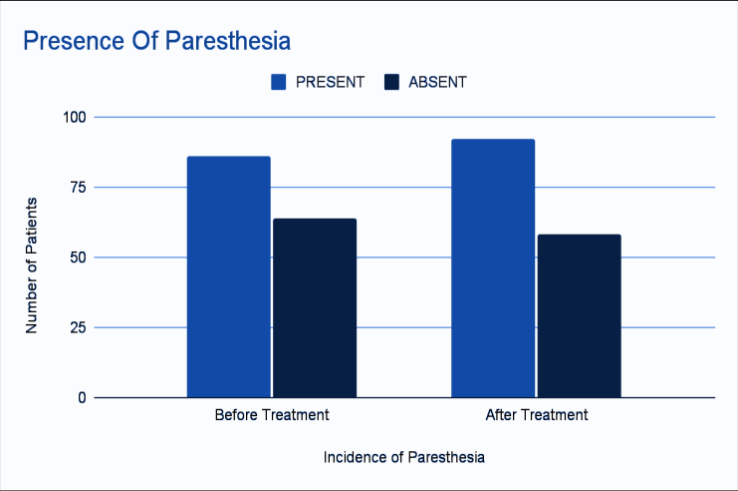
Bar Diagram of Incidence of Paresthesia

The crosstabs among the two groups showed that there were 86 cases of paresthesia present both before and after treatment, 58 cases where paresthesia was absent both before and after treatment, and 6 cases where paresthesia was absent before treatment but present after treatment. The descriptive statistical analysis was conducted using binomial distribution and the McNemar Test. A significant difference was observed between the two groups, with a p-value of 0.031 (p < 0.05) (Table 3) (Figure 3). The overall incidence of IAN deficit was 57.33% before treatment and 61.33% after treatment.

## Discussion:

Mandibular fractures are a common consequence of facial trauma, often resulting in injury to the inferior alveolar nerve (IAN) and causing sensory disturbances to the lower lip and chin [4]. The IAN can be damaged within the mandibular canal or after its exit from the mental foramen, either due to direct trauma or surgical reduction and fixation of the fracture. Posttraumatic disorders of the IAN significantly impact patients' quality of life [5], yet there is limited documentation on the incidence and patterns of posttraumatic IAN sensory disturbances in the maxillofacial trauma literature [6] [7]. Therefore, it is important to investigate the incidence of IAN injury in trauma patients with mandibular fractures during the presentation. A study reported hypoesthesia of the inferior alveolar nerve in 27% of patients preoperatively and in 46% of patients postoperatively [8]. Additionally, another study reported higher rates of incidence of inferior alveolar nerve injury after treatment (53.8%), compared to before treatment (33.7%) [9]. these studies provide findings consistent with the results of this study. The most common complications of open reduction and internal fixation include pain, swelling, trismus, paresthesia, and plate removal [10,11, 12]. Additionally, neurosensory disturbances of the IAN can present as loss of vitality in affected teeth, with 81% of the affected teeth showing no response [13]. Studies have demonstrated that closed reduction can help prevent post-treatment nerve injury [14]. While anatomical variations in the course of the inferior alveolar nerve exist, there are no studies presenting these variations in IAN injuries [15, 16, 17, 18, 19].

## Conclusions:

The overall incidence of inferior alveolar nerve (IAN) injuries was 57.33%, with neurosensory disturbances reported in 86 patients before treatment and 61.33% reported in 92 patients after treatment. The mean age was 34.3 years, and the majority of patients were male (56%). Assaults were the leading cause of mandibular fractures (38%), followed by road traffic accidents (36%) and falls (26%). The highest incidence of IAN deficit was observed in patients with the body of the mandible fracture before treatment, which may be due to the injury caused post-trauma. These findings highlight the importance of promptly identifying and managing IAN injuries to minimize long-term consequences.

## Figures and Tables

**Table 1:** Demographic representation of the population

	**Mean Age**	**Male**	**Female**
N (150)	34.3 ±7.67	97 (64.67%)	53 (35.33%)
The table displayed provides the demographic information of the participants included in the study. The mean age of the subjects was 34.03 ± 7.67 with a gender distribution of 64.67% of the patients being male and 35.33% being female.

**Table 2:** IAN injury and site of fracture before treatment

**Cause**	**Patients (N)**			**Percentage**
	With IAN deficit	Without IAN deficit	Total	
Ramus	0	5	5	3.33%
Angle	37	26	63	42%
Body	49	33	82	54.67%
Total	86	64	150	100

**Table 3:** Descriptive statistical analysis

**GroupA** and **GroupB**
GroupA	GroupB
	Absent	Present
Absent	58	6
Present	0	86
Test Statistics^a^
GroupA and GroupB
N	150
Exact Sig. (2-tailed)	.031^b^
